# Epidemic Ague, or Fainting Fever of Persia

**Published:** 1844-07

**Authors:** 


*Epidemic Ague, or Fainting Fever of Persia.*—Dr. Bell, physician to her Majesty's mission at Persia, has published an account of this disease, with which four-fifths of the inhabitants of Teheran were attacked, and above one-sixteenth of the whole population died of it. It presented some points of analogy with the cholera asphyxia in its nature and habits, and pursued the same north-westerly course which that epidemic took some years since, when it visited Europe. Although dangerous when left to maturity, it is most amenable to treatment. It is essentially a quotidian ague. The following are the principal modes in which it produces its dangerous or fatal effects: general venous congestion, and consequently impeded action of the heart and cessation of the circulation, sudden effusion into the lungs, exudation from the capillaries generally into the cellular tissue, diseased spleen, constantly recurring ague, general dropsy and failure of the powers of life. There may have been other causes, but these Dr. Bell was unable to note, from not having been able to obtain post-mortem examinations. There are several varieties of the disease, more or less marked in their character, the more severe merging into true cholera. The treatment consisted in the exhibition of quinine with iron and sulphate of magnesia, and in bleeding from the arm, where requisite. The blood generally presented characters similar to those noticed in cholera.—*London Med. Times.*

